# Supplementary material for: Time-integrated BMP signaling determines fate in a stem cell model for early human development
Source: Nat Commun. 2024 Feb 17;15:1471. doi: 10.1038/s41467-024-45719-9 (PMC10874454; doi:10.1038/s41467-024-45719-9)
Supplement: Supplementary file 1 — Supplementary Information [file 41467_2024_45719_MOESM1_ESM.pdf]

Supplementary information

Time-integrated BMP signaling determines fate in a stem cell model for early human development

Seth Teague[1], Gillian Primavera[1], Bohan Chen[2], Zong-Yuan Liu[3], LiAng Yao[3], Emily Freeburne[3], Hina Khan[3], Kyoung Jo[3], Craig Johnson[3], Idse Heemskerk[1,2,3,4,5]*

1. Department of Biomedical Engineering, University of Michigan, Ann Arbor, Michigan
2. Department of Computational Medicine and Bioinformatics, University of Michigan Medical School, Ann Arbor, Michigan
3. Department of Cell and Developmental Biology, University of Michigan Medical School, Ann Arbor, Michigan
4. Center for Cell Plasticity and Organ Design, University of Michigan Medical School, Ann Arbor, Michigan
5. Department of Physics, University of Michigan, Ann Arbor, Michigan

*for correspondence: iheemske@umich.edu

Supplementary figures

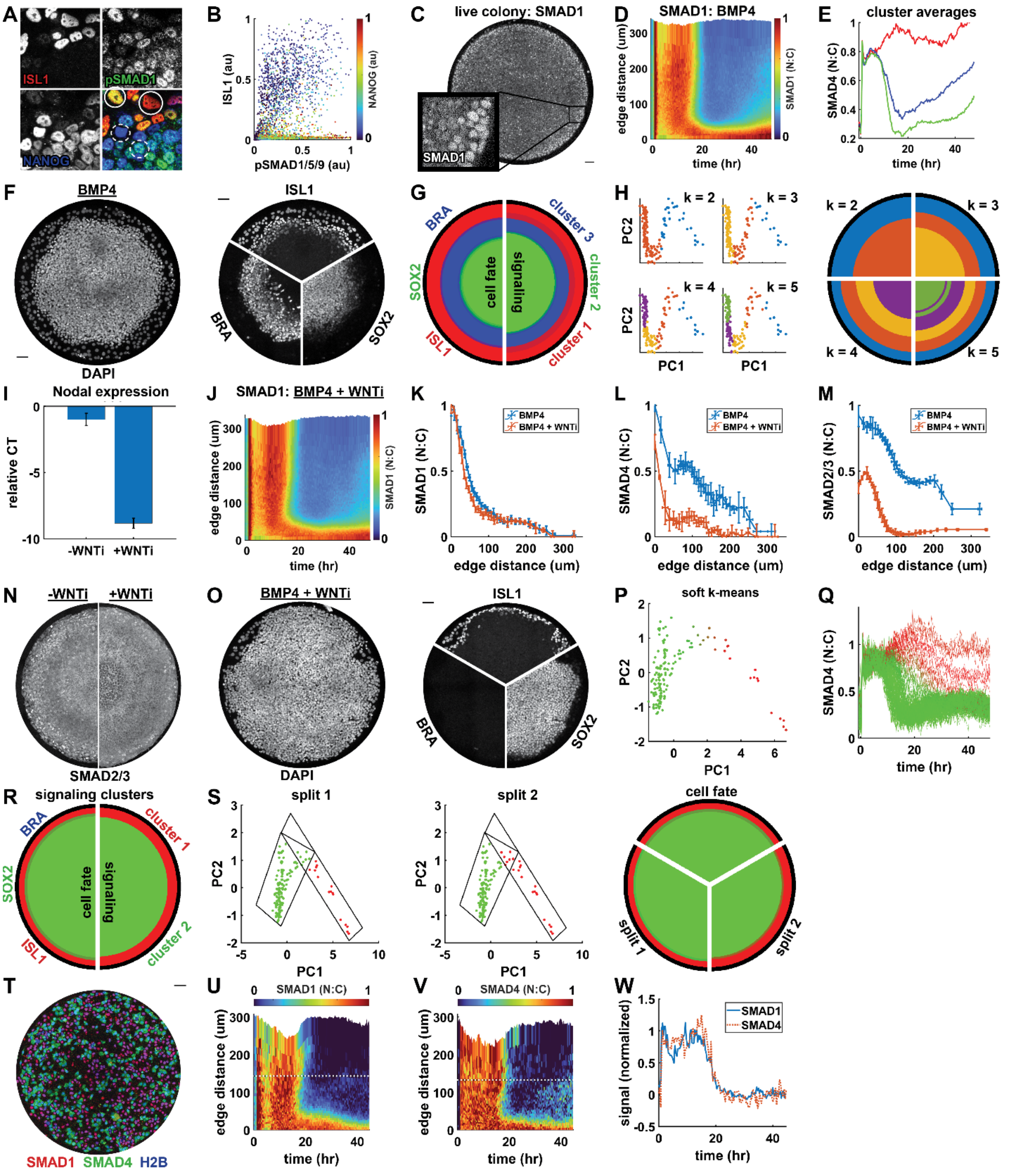

Supplementary Figure 1: (A) Detail of micropatterned colony stained for pSMAD1/5/9, ISL1, and NANOG after treatment with BMP + WNTi (IWP2) showing both amnion-like cells (solid circles) and pluripotent cells (circles) with high and low signaling levels. **(B)** Quantification confirms low correlation between final signaling and fate. (**C**) RFP::SMAD1 cells at t = 30 hours after treatment with BMP4, showing stronger nuclear localization of SMAD1 at the colony edge. (**D**) Kymograph of mean SMAD1 signaling of N=5 BMP4-treated colonies. (**E**) Means of signaling history clusters in Fig.1H. (**F**) Separate DAPI, ISL1, SOX2, and BRA images for colonies in Fig. 1J. (**G**) Average cell fate marker profile (left) versus signaling history clusters (right) for BMP4-treated colonies. (**H**) Different cluster numbers visualized on PCA plot (left) and spatially (right). (**I**) RT-qPCR shows large reduction in Nodal expression in

micropatterned colonies treated with WNTi at 42h. Error bars: standard deviation over technical triplicates. **(J)** Kymograph of SMAD1 dynamics in colonies treated with BMP4 and WNTi. (**K-M**) Spatial profile at 42h with or without WNTi for GFP::SMAD4 (K), RFP::SMAD1 (L), and anti-SMAD2/3 (M). (**N**) SMAD2/3 staining with or without WNTi. (**O**) Separate DAPI, ISL1, SOX2, and BRA images corresponding to the colonies shown in Fig. 1N.**(P)** Scatterplot of the first two PCs of radially averaged signaling histories, colored for soft k-means cluster assignment as in Fig 1G. **(Q)** Plot of radially averaged signaling histories colored for cluster assignment in (P). **(R)** Cell fate pattern with WNTi (left) versus spatial pattern of signaling clusters in (P) (right). (**S**) Example of different ways to assign the signaling histories corresponding to the 'elbow' of the PCA plot in colonies treated with BMP4 + WNTi and resulting cluster patterns compared to the cell fate pattern. (**T**) Overlay of RFP::SMAD1 (red), GFP::SMAD4 (green) and H2B (blue) images in a single colony with mixed SMAD1 and SMAD4 cells (see methods). (**U-V**) Kymographs of SMAD1 (U) and SMAD4 (V) nuclear localization averaged over the same N=4 colonies. (**W**) Cross section through the kymographs in (U) and (V) where indicated with a white dashed line. Scale bars 50um. Source data are provided in a Source Data file.

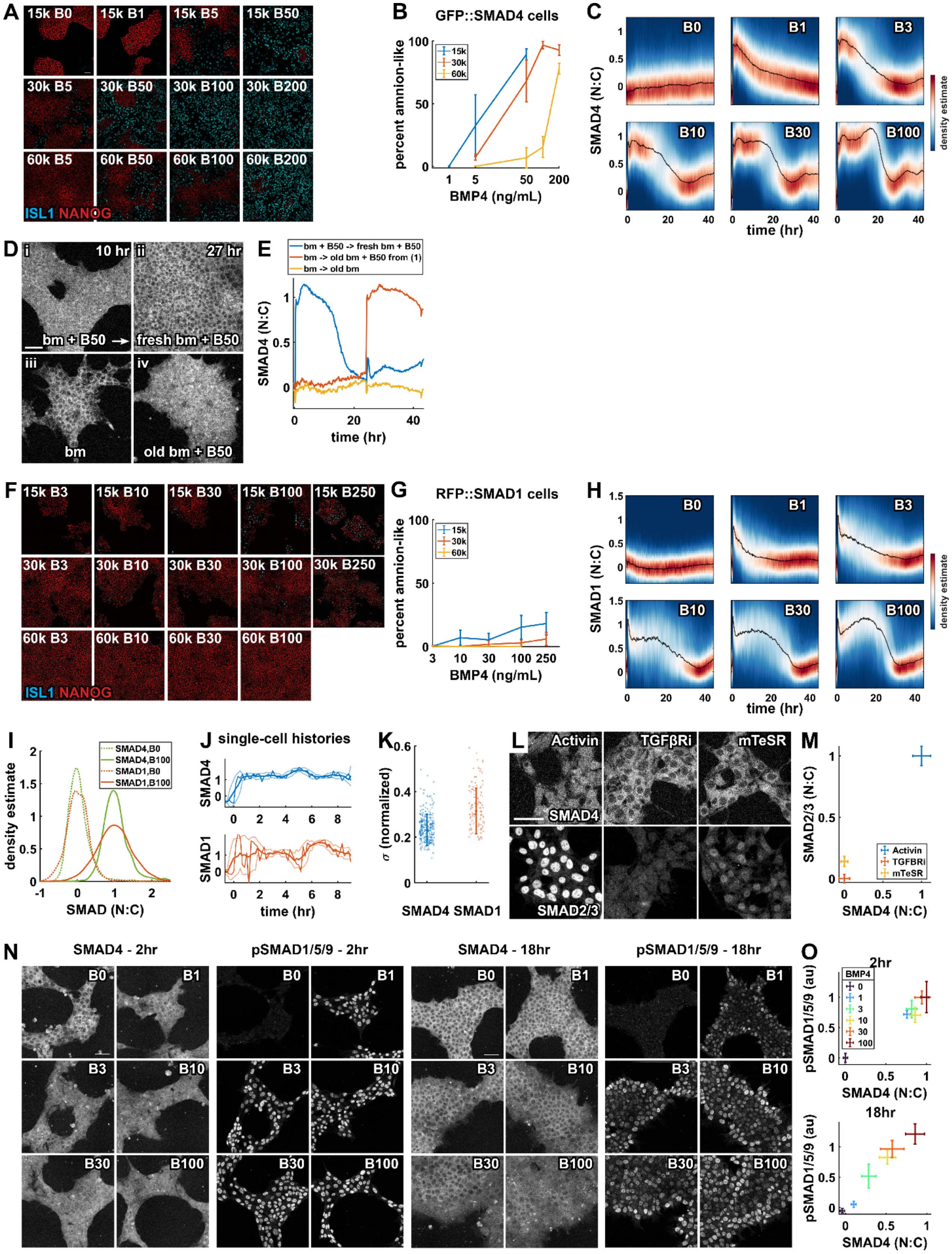

Supplementary Figure 2: (**A**) Immunofluorescence staining for NANOG and ISL1 in RUES2 GFP::SMAD4 cells for different conditions, first number is density, e.g. 15k = 15,000 cells / cm^2, second is BMP4 dose, e.g. B1 = 1ng/ml BMP4. **(B)** Quantification of (A). **(C)** Heatmap of SMAD4 signaling distribution over time corresponding for different BMP4 doses and 30k cells/cm^2. (**D**) Media change with fresh BMP for cells initially treated with 50ng/ml BMP4 (i)

shows no response (ii), while transfer of old media to untreated cells (iii) induces strong BMP response (iv). 'bm' = base media. (**E**) Quantification of SMAD4 corresponding to conditions in (D) and control for media change without BMP4. (**F**) Immunofluorescence staining in RUES2 RFP::SMAD1 cells for different conditions as in (A). **(G)** Quantification of (F). **(H)** Heatmap of SMAD1 signaling distribution over time for different BMP4 doses and 30k cells/cm^2. (**I**) Kernel density estimate of SMAD4 and SMAD1 signaling readouts in untreated cells and cells treated for two hours 100 ng/mL BMP4. Data is normalized so average signaling in B100 = 1 and average signaling in B0 = 0. (**J**) Example single cell signaling histories in GFP::SMAD4 and RFP::SMAD1 cells tracked over the first 10 hours of response to 10 ng/mL BMP4, normalized similarly to (I). The raw readout is shown as a thin solid line, average signaling in a rolling 1-hour window is shown as a thicker line, and the average +/- one standard deviation within the 1-hour window is shown as semitransparent lines. (**K**) Scatterplot of the median standard deviation in time of signaling in individual tracked SMAD1 and SMAD4 cells, with error bars showing the mean and standard deviation over tracks. (**L**) Example images of live GFP::SMAD4 and IF for SMAD2/3 after 1.5 hours of treatment with Activin A, SB431542 (TGFβRi), or neither (mTeSR). (**M**) Quantification of (L). (**N**) Images of live GFP::SMAD4 and IF for pSMAD1/5/9 in the same cells after fixation at 2 and 18 hours after BMP4 treatment. Treatment conditions are the same as in (C). (**O**) Quantification of mean +/- standard deviation over N = 7 images in (N). All scale bars = 50 µm. Source data are provided in a Source Data file.

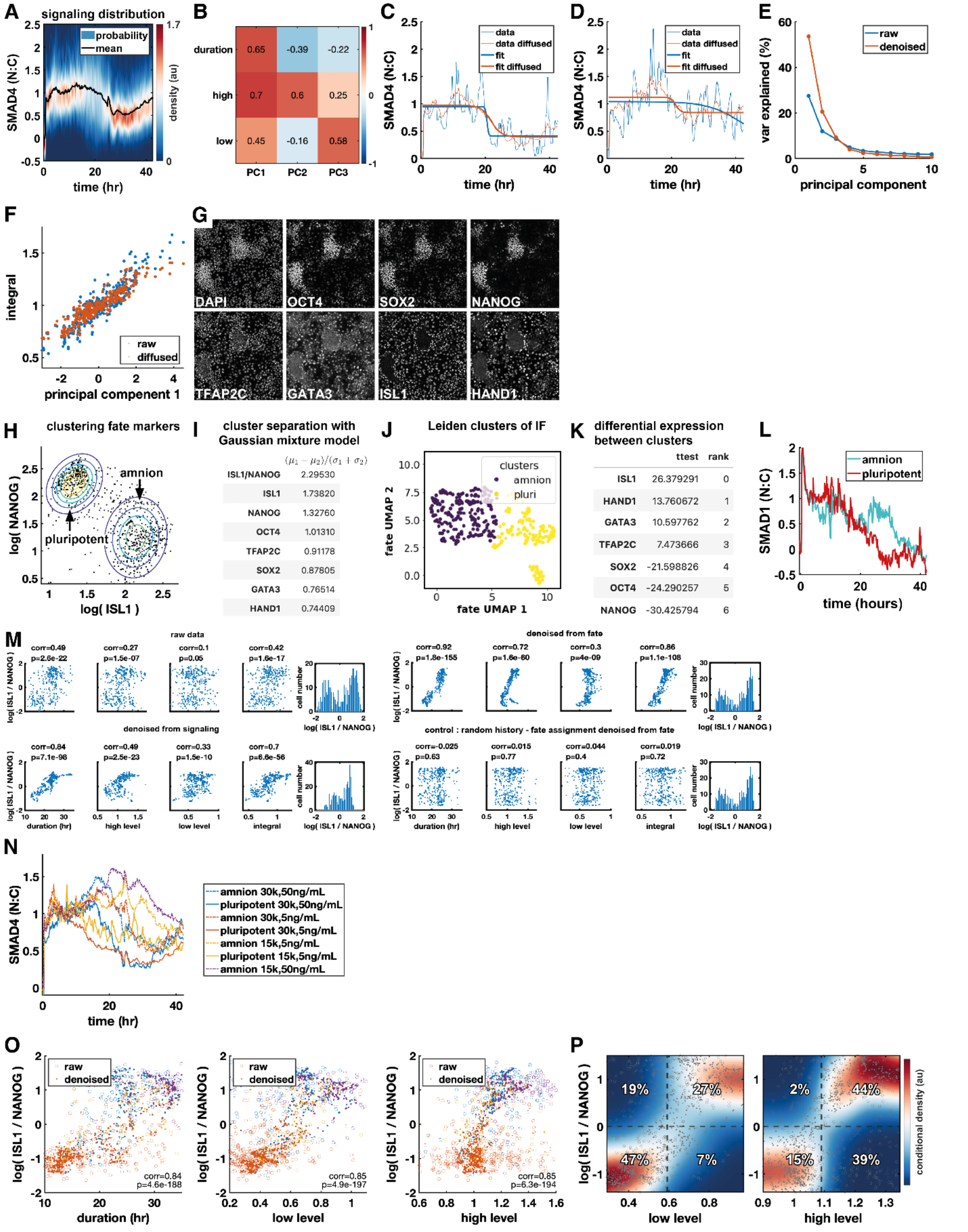

Supplementary Figure 3: **(A)** Heatmap of signaling distribution over time corresponding to Fig. 3AB. **(B)** Correlation of features and principal components (PCs) before denoising. **(C-D)** Example signaling histories before and after denoising with MAGIC, with sigmoid fits to raw and denoised data. **(E)** Variance explained in signaling distribution from (A) by the first 10 PCs for raw and denoised signaling histories. (**F**) Scatterplot of signal integral against principal component 1, with and without denoising. (**G**) Representative single-channel IF images showing expression of all 7 stained genes in the same field of view. **(H)** Contour plot of a two-component Gaussian mixture model overlaid on a

scatterplot of ISL1 vs. NANOG. (**I**) Table of values of a measure of cluster separation. The marginal distribution of the 7D Gaussian mixture model (GMM) is taken along each axis indicated and the separation of clusters along that direction is taken as the ratio of the difference in the means of the two GMM components to the sum of their standard deviations. A higher value indicates better separation. (**J**) UMAP plot showing the separation of cells into two clusters with Leiden clustering. (**K**) Table of differential expression of each marker between the Leiden clusters, showing highest absolute values for ISL1 and NANOG. (**L**) mean RFP::SMAD1 signaling in amnion and pluripotent cells. (**M**) Scatterplots of log(ISL1 / NANOG) and signaling features under various denoising schemes for data in Fig. 3A-J. (**N**) Fig. 3L colored by condition. **(O)** Scatter plots of signaling features vs. log(ISL1/NANOG) colored for condition with and without denoising for data in Fig. 3K-P. Color is by condition as in Fig 3K. **(P)** Heatmap of kernel density estimate after denoising of conditional distributions of log(ISL1 / NANOG) with respect to low level and high level of signaling, overlaid with a scatterplots of data points before (circles) and after denoising (dots). Dashed lines show separation of cells into amnion-like and pluripotent based on log(ISL1 / NANOG) or on signaling features. The percentage of cells in each quadrant is indicated, with correct assignments in the top right and bottom left quadrant of each heatmap. Source data are provided in a Source Data file.

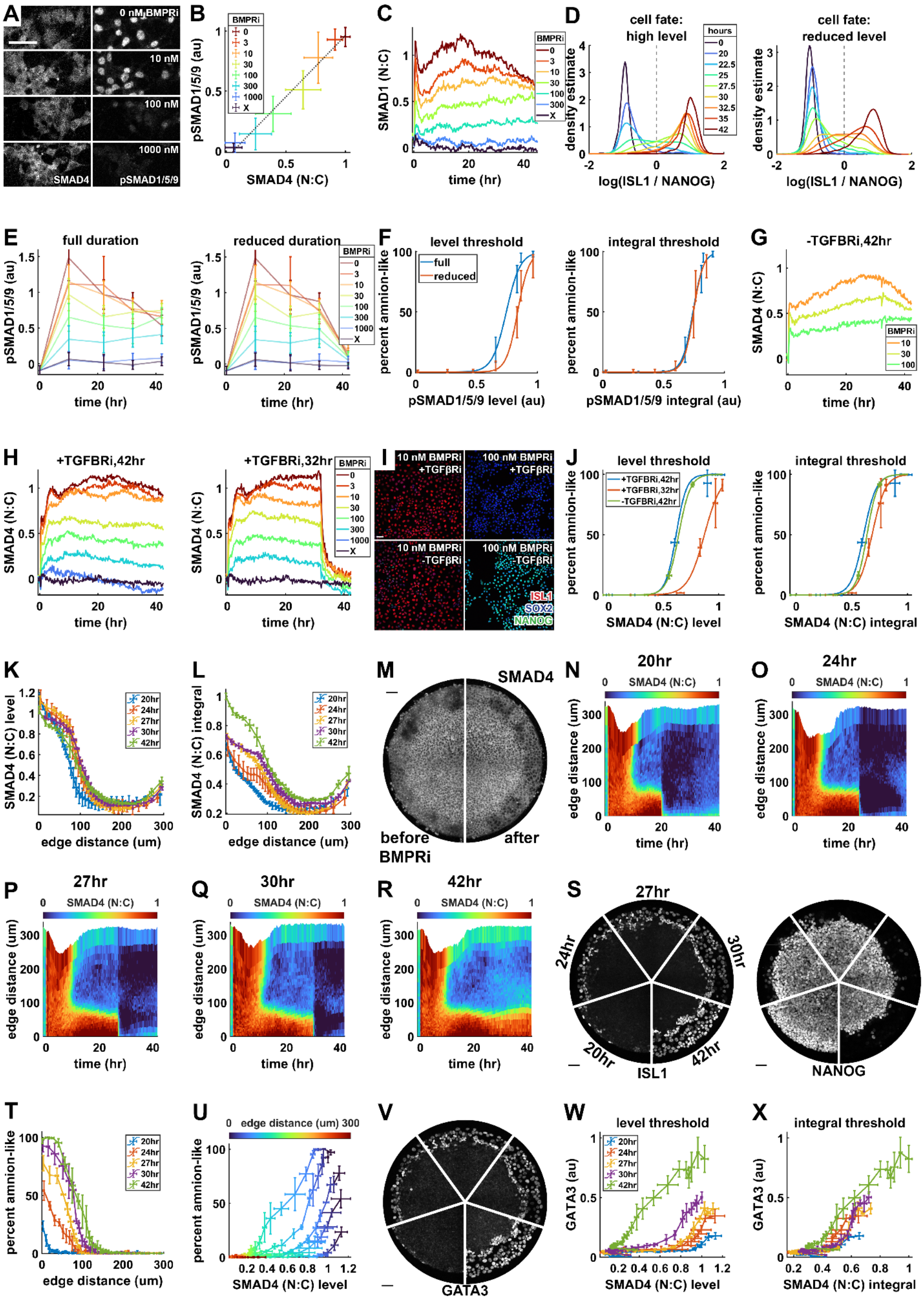
A
0 nM BMPRi
10 nM
100 nM
1000 nM
SMAD4
pSMAD1/5/9
B
pSMAD1/5/9 (au)
SMAD4 (N:C)
C
SMAD1 (N:C)
time (hr)
D
cell fate: high level
cell fate: reduced level
density estimate
log(ISL1 / NANOG)
hours
E
full duration
reduced duration
F
level threshold
integral threshold
percent amnion-like
full
reduced
pSMAD1/5/9 level (au)
pSMAD1/5/9 integral (au)
G
-TGFBRi,42hr
SMAD4 (N:C)
H
+TGFBRi,42hr
+TGFBRi,32hr
BMPRi
I
10 nM BMPRi +TGFβRi
100 nM BMPRi +TGFβRi
10 nM BMPRi -TGFβRi
100 nM BMPRi -TGFβRi
ISL1
SOX2
NANOG
J
level threshold
integral threshold
+TGFBRi,42hr
+TGFBRi,32hr
-TGFBRi,42hr
SMAD4 (N:C) level
SMAD4 (N:C) integral
K
SMAD4 (N:C) level
edge distance (um)
L
SMAD4 (N:C) integral
M
SMAD4
before BMPRi
after
N
20hr
O
24hr
edge distance (um)
P
27hr
Q
30hr
R
42hr
S
27hr
24hr
30hr
20hr
42hr
ISL1
NANOG
T
percent amnion-like
U
edge distance (um)
SMAD4 (N:C) level
V
GATA3
W
level threshold
GATA3 (au)
X
integral threshold

Supplementary Figure 4: (A) GFP::SMAD4 and pSMAD1/5/9 images of sparsely seeded cells treated with 100 ng/ml BMP4 and different doses of LDN193189 (BMPRi, doses in nM). (**B**) Linear fit to average pSMAD1/5/9 and GFP::SMAD4 signaling in (A). 'X': untreated cells. (**C**) RFP::SMAD1 nuclear level in sparse cells treated with high BMP4 and titrated BMPRi, as in (A). (**D**) Kernel density estimate of the log(ISL1/NANOG) distribution corresponding to 4E. (**E**) pSMAD1/5/9 immunofluorescence levels at different times in wild type hESCs under conditions like 4E. Error bars: standard deviation over images pooled from two independent experiments. (**F**) Differentiation versus pSMAD1/5/9 level and integral for data from (E). (**G-H**) Average SMAD4 signaling (nuclear:cytoplasmic ratio) in conditions like 4E with and without addition of the TGFβ receptor inhibitor SB431542 (TGFβRi). (**I**) Immunofluorescence for ISL1, SOX2, and NANOG in selected conditions from (G,H), showing downregulation of NANOG but not SOX2 with low BMP signaling in the presence of TGFβRi. Red = ISL1+/SOX2-/NANOG-; Blue = ISL1-/SOX2+/NANOG-; Cyan = ISL1-/SOX2+/NANOG+. (**J**) Amnion-like differentiation vs. SMAD4 signaling level and integral for data from (G) and (H). **(KL)** Average SMAD4 signaling level before BMP inhibition (K) and integral (L) versus distance from the colony edge for different durations of BMP signaling. (**M**) GFP::SMAD4 for a colony treated with 200ng/ml BMP4 shown before (29h, left) and after BMP signaling inhibition (31h, right). **(N-R)** Kymographs of average SMAD4 signaling in N=3 micropatterned colonies each for five signaling durations. **(S)** ISL1 and NANOG immunofluorescence stains of colonies exposed to different durations of BMP signaling. **(T)** Percentage of differentiated cells (ISL1+NANOG-) versus distance from the colony edge for different durations of BMP signaling. **(U)** Percent amnion differentiation vs. level of BMP signaling as in Fig. 4M, colored for distance from the colony edge. **(V)** Representative IF images showing the spatial extent of GATA3 expression in micropatterned colonies exposed to different durations of BMP signaling. **(WX)** GATA3 expression in radial bins as a function of SMAD4 signaling level (W) and integral (V) for different signal durations. Error bars: standard deviation over N = 3 colonies. Scale bars 50um. Source data are provided in a Source Data file.

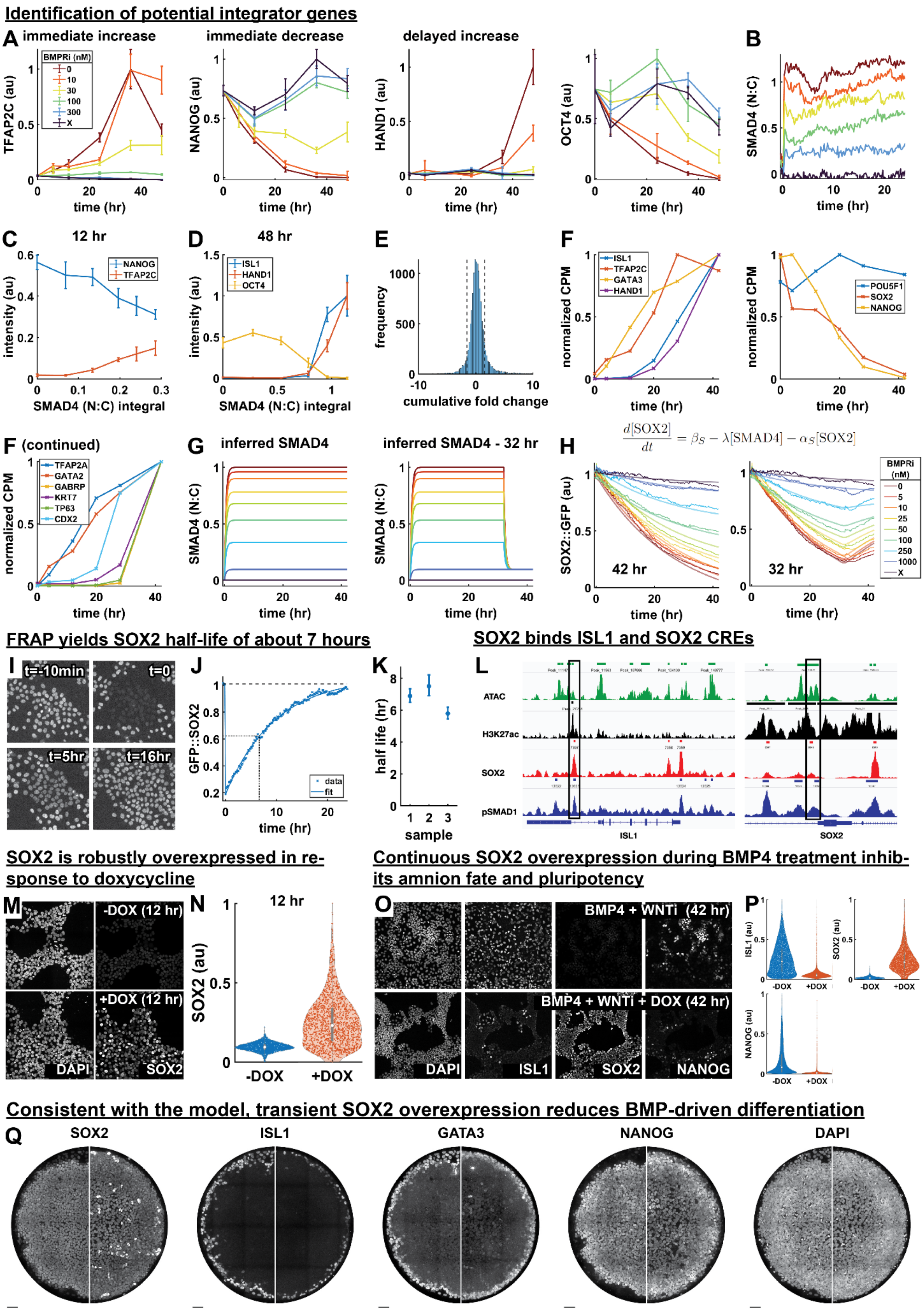
Identification of potential integrator genes
A immediate increase
immediate decrease
delayed increase
B
TFAP2C (au)
NANOG (au)
HAND1 (au)
OCT4 (au)
SMAD4 (N:C)
BMPRi (nM)
0
10
30
100
300
X
time (hr)
C 12 hr
D 48 hr
E
F
intensity (au)
NANOG
TFAP2C
ISL1
HAND1
OCT4
SMAD4 (N:C) integral
frequency
cumulative fold change
normalized CPM
ISL1
TFAP2C
GATA3
HAND1
POU5F1
SOX2
NANOG
F (continued)
G inferred SMAD4
inferred SMAD4 - 32 hr
H
$\frac{d[\mathrm{SOX2}]}{dt} = \beta_S - \lambda[\mathrm{SMAD4}] - \alpha_S[\mathrm{SOX2}]$
TFAP2A
GATA2
GABRP
KRT7
TP63
CDX2
SMAD4 (N:C)
SOX2::GFP (au)
42 hr
32 hr
BMPRi (nM)
0
5
10
25
50
100
250
1000
X
FRAP yields SOX2 half-life of about 7 hours
SOX2 binds ISL1 and SOX2 CREs
I t=-10min
t=0
t=5hr
t=16hr
J
GFP::SOX2
data
fit
K
half life (hr)
sample
L
ATAC
H3K27ac
SOX2
pSMAD1
ISL1
SOX2
SOX2 is robustly overexpressed in response to doxycycline
Continuous SOX2 overexpression during BMP4 treatment inhibits its amnion fate and pluripotency
M
-DOX (12 hr)
+DOX (12 hr)
DAPI
SOX2
N 12 hr
SOX2 (au)
-DOX
+DOX
O
BMP4 + WNTi (42 hr)
BMP4 + WNTi + DOX (42 hr)
DAPI
ISL1
SOX2
NANOG
P
ISL1 (au)
SOX2 (au)
NANOG (au)
-DOX
+DOX
Consistent with the model, transient SOX2 overexpression reduces BMP-driven differentiation
Q
SOX2
ISL1
GATA3
NANOG
DAPI

Supplementary Figure 5: (**A**) Normalized expression of TFAP2C, NANOG, HAND1, and OCT4 over time for different signaling levels, measured with time-series immunofluorescence (IF). Error bars are standard deviation across N = 6 replicate images. (**B**) Average SMAD4 dynamics measured in the treatment conditions for which time-series IF was performed. Legend in (A). (**C**) NANOG and TFAP2C expression at 12 hours, plotted against SMAD4 signaling integral. (**D**) ISL1, HAND1, and OCT4 expression at 48 hours, plotted against SMAD4 signaling integral, showing switch-like reliance. (**E**) Histogram of the cumulative log2 fold change over all genes in the time-series bulk RNA seq data. Genes with a fold change between the two dotted lines were not included in hierarchical clustering or subsequent analysis. (**F**) Expression over time for example genes measured with bulk RNA seq. Expression of amnion (left) and pluripotency (middle) genes that were also measured with time series IF, and additional amnion and trophectoderm genes (right). (**G**) Idealized SMAD4 dynamics used as input to the ODE model, with level inferred from data in fig 4. (**H**) GFP::SOX2 dynamics over the course of 42 hours of differentiation with indicated treatments applied for 42 (left) or 32 (right) hours, overlaid with fits of the simple ODE model described by the equation above it for SOX2, and the equation for ISL1 as in Fig. 6D. (**I-K**) GFP::SOX2 fluorescence recovery after photobleaching: representative images (I), example intensity recovery curve (J), and half-life measured in three replicate fields of view (K). (**L**) ChIP-seq data showing SOX2 and pSMAD1 binding peaks coinciding with H3K27ac and ATAC peaks marking active ISL1 enhancers and SOX2 promoter. (**M**) Representative IF images showing expression of SOX2 after 12 hours in pluripotency conditions with or without doxycycline. (**N**) Violin plot of SOX2 expression after 12 hours with or without doxycycline in pluripotency conditions. (**O**) Representative IF images showing ISL1, SOX2, and NANOG expression after 42 hours of treatment with BMP4 + WNTi with or without doxycycline. (**P**) Violin plots of ISL1, NANOG, and SOX2 expression with or without doxycycline in differentiation conditions. (**Q**) Individual channels corresponding to Fig. 6I. Scale bars 50um. Source data are provided in a Source Data file.

Supplementary Table 1: Cell signaling reagents

Reagent	Nickname	Vendor, cat #	Dose	Function
rhBMP4	BMP4	R&D Systems, 314BP/CF	See figures	Activate BMP pathway
IWP 2	WNTi	ApexBio, A3512-5	5 µM	Block Wnt secretion
LDN-193189	BMPRi	MedChemExpress, HY-12071	See figures	Inhibit BMP receptors
SB431542	TGFβRi	ApexBio, A8249	10 µM	Inhibit TGFβ receptors
rhNOGGIN	NOGGIN	R&D Systems, 6057-NG-025/CF	250 ng/mL	Inhibit BMP signaling
rActivin A (human/mouse/rat)	Activin A	R&D Systems, 338AC050	100 ng/mL	Activate Activin/Nodal pathway

Supplementary Table 2: Primary antibodies used for immunofluorescence

Protein	Species	Dilution	Catalog #	Vendor
ISL1	Mouse	1:200	39.4D5	DSHB
SOX2	Rabbit	1:200	3579S	Cell Signaling Technology
NANOG	Goat	1:100	AF1997	R&D Systems
HAND1	Goat	1:200	AF3168	R&D Systems
GATA3	Rabbit	1:800	5852S	Cell Signaling Technology
TFAP2C	Mouse	1:150	SC-12762	Santa Cruz Biotechnology
OCT3/4	Mouse	1:400	611202	BD Biosciences
pSMAD1/5/9	Rabbit	1:100	13820S	Cell Signaling Technology
SMAD2/3	Mouse	1:100	610843	BD Biosciences
BRA	Goat	1:300	AF2085	R&D Systems

Supplementary Table 3: Secondary antibodies

Protein	Species	Dilution	Catalog #	Vendor
Alexa Fluor 488 anti-mouse	Donkey IgG	1:500	A21202	ThermoFisher Scientific
Alexa Fluor 555 anti-rabbit	Donkey IgG	1:500	A31572	ThermoFisher Scientific
Alexa Fluor 647 anti-goat	Donkey IgG	1:500	A21447	ThermoFisher Scientific

Alexa Fluor 647 anti-mouse	Donkey IgG	1:500	A31571	ThermoFisher Scientific
Alexa Fluor 647 anti-rabbit	Donkey IgG	1:500	A31573	ThermoFisher Scientific
Alexa Fluor 555 anti-goat	Donkey IgG	1:500	A21432	ThermoFisher Scientific

Supplementary Note 1: Mathematical model of BMP-SMAD4 integration

Minimal model for SOX2 regulation by BMP

We aimed to develop a mathematical model to explain how a simple gene regulatory network (GRN) could integrate BMP-SMAD4 signaling in time. In the simplest model, the expression of an integrator gene directly reflects the time integral of SMAD4 signaling. This is analogous to looking for genes with a rate of change that is a linear function of BMP-SMAD4 signaling, i.e., those that can be modeled with an ordinary differential equation (ODE) approximately as

$$\frac{d[\mathrm{GENE}]}{dt} = \lambda[\mathrm{SMAD4}](t),$$

where [GENE] gives the concentration of protein and $[\mathrm{SMAD4}](t)$ is the (time-varying) level of BMP signaling. Integration of the above results in production of the gene product that is directly proportional to the signaling integral, i.e.,

$$[\mathrm{GENE}](t) = \lambda \int_0^t [\mathrm{SMAD4}](\tau)\, d\tau + [\mathrm{GENE}]_0,$$

where the initial concentration of protein is given by $[\mathrm{GENE}]_0 = [\mathrm{GENE}](0)$. More generally, we can allow an additional constant term β for constitutive production, so that protein production remains a linear function of signaling. To account to protein turnover we add a decay term that is proportional to the current concentration of protein. The ODE model then becomes

$$\frac{d[\mathrm{GENE}]}{dt} = \beta + \lambda[\mathrm{SMAD4}](t) - \alpha[\mathrm{GENE}].$$

Our screen for genes for which the rate of change is linear with SMAD4 signaling level found SOX2 to be a promising candidate, as measured at the protein level with immunofluorescence, and at the transcript level with RNA sequencing. If SOX2 is our integrator, its dynamics should roughly follow integrated SMAD4 signaling, and it should repress late-response amnion genes so that they are expressed only if SOX2 goes below a threshold level. We first aim to determine whether SOX2 dynamics can be plausibly modeled with the dynamics described above. SOX2 is negatively regulated by BMP signaling, so we rewrite the ODE as

$$\frac{d[\mathrm{SOX2}]}{dt} = \beta - \lambda[\mathrm{SMAD4}](t) - \alpha[\mathrm{SOX2}], \tag{1}$$

where λ is taken to be positive. In the absence of BMP signaling, $[\mathrm{SMAD4}] = 0$ and SOX2 expression tends to a steady-state value of β/α balancing constitutive production and decay. A model of the form

$$\frac{dy}{dt} = f(t) - \alpha y,$$

where f is a function of t but not y has the solution

$$y(t) = e^{-\alpha t} \int_0^t f(t) e^{\alpha\tau}\, d\tau + y_0 e^{-\alpha t}.$$

So the concentration of SOX2 is modeled by

$$[\mathrm{SOX2}](t) = \beta/\alpha + ([\mathrm{SOX2}]_0 - \beta/\alpha)e^{-\alpha t} - \lambda e^{-\alpha t} \int_0^t [\mathrm{SMAD4}](\tau) e^{\alpha\tau}\, d\tau.$$

We assume that SOX2 is at or very near the steady state β/α in pluripotency maintenance conditions prior to stimulation of BMP signaling, i.e. $[\text{SOX2}]_0 = \beta/\alpha$. We further normalize this pretreatment expression level to one, so the expression for SOX2 as a function of time reduces to

$$[\text{SOX2}](t) = 1 - \lambda e^{-\alpha t} \int_0^t [\text{SMAD4}](\tau) e^{\alpha \tau} \, d\tau. \tag{2}$$

We see that the level of SOX2 protein reflects an exponentially-weighted integral of SMAD4 signaling, which approximates an exact integral of SMAD4 signaling if t is much smaller than $1/\alpha$ (where $e^{-\alpha t} \approx e^{\alpha t} \approx 1$).

To fit the model we measured GFP::SOX2 dynamics in hPSCs for 42 hours of BMP4-driven differentiation in conditions in which we could control the level and duration of signaling. Briefly, as described in the main text, we treated sparsely seeded hPSCs with a high dose of BMP4 to ensure uniformly high response, and controlled the signaling level via titration of a BMP receptor inhibitor (BMPRi). To control the duration, we shut down signaling by removing BMP4 and adding a high dose of BMPRi. We measured GFP::SOX2 dynamics at a range of signaling levels with durations of 42 and 32 hours (Supplementary Fig.5H).

We approximated input SMAD4 dynamics as flat with levels measured in cells expressing GFP::SMAD4 in the same treatment conditions as the GFP::SOX2 cells (Fig.4; Supplementary Fig.5G). We confirmed the linear relationship between SMAD4 signaling level and rate of SOX2 decay with a linear fit to the first 16 hours of GFP::SOX2 dynamics in each condition (Fig.6A). To determine the values of the model parameters, we collected values of SOX2, SMAD4, and the slope of SOX2, averaged over short time windows to reduce the effect of measurement noise. We fit a plane defined by equation (1) to our to our measured values of $d[\text{SOX2}]/dt$, [SOX2], and [SMAD4] to find the values of β, λ, and α.

Numerically integrating the model with the fitted parameters and with measured input SMAD4 levels, we saw generally good agreement with measured SOX2, but later in the course of differentiation in conditions with the highest levels of signaling, SOX2 is higher than expected (Supplementary Fig.5H, red and orange curves). In particular, when signaling is inhibited, the model predicts strong recovery of SOX2 in conditions where SOX2 has been highly downregulated but this does not occur (Supplementary Fig.5H, right), while lower signaling conditions do see strong recovery.

Modeling differentiation

We hypothesized that the failure of SOX2 to recover after signaling shutdown in conditions with higher initial signaling reflected differentiation, since SOX2 is repressed in differentiated cells and would not be expected to recover. Differentiation is accompanied by upregulation of late-response amnion genes. We used ISL1 as a representative example of that class of genes and measured ISL1 expression in GFP::SOX2 genes after 42 hours with immunofluorescence.

We modeled differentiation by assuming SOX2 directly represses ISL1 expression, which is the simplest way in which SOX2 can act as our integrator gene. We additionally modeled repression of SOX2 by ISL1 so that SOX2 expression is further downregulated once ISL1 begins to be expressed. This fits the paradigm of mutually inhibitory regulatory programs specifying distinct cell fates that are widespread in development [1], [2]. To implement this mutual repression mathematically, we

took each gene to act on the other with Hill function dependence:

$$\frac{d[\text{SOX2}]}{dt} = (\beta_S - \lambda_S[\text{SMAD4}]) \cdot \frac{K_{SI}^{n_S}}{K_{SI}^{n_S} + [\text{ISL1}]^{n_S}} - \alpha_S[\text{SOX2}], \tag{3}$$

$$\frac{d[\text{ISL1}]}{dt} = \frac{\lambda_I[\text{SMAD4}]}{1 + ([\text{SOX2}]/K_{SI})^{n_I}} - \alpha_I[\text{ISL1}]. \tag{4}$$

In the above equations, the parameters K describe the threshold for 50% inhibition of one gene by the other and n describes the steepness of the Hill function. In our time series expression data for ISL1 (Fig.5A, Supplementary Fig.5F), we see that it remains close to zero until 20-24 hours, when expression rapidly switches on, suggesting that there is a sharp threshold for regulation of ISL1. We therefore modeled repression of ISL1 by SOX2 with a switch-like Hill function by setting $n_I = 4$. On the other hand, repression of SOX2 by the amnion transcriptional program appears more graded, and we take $n_S = 2$.

We used simulated annealing to fit equations (3) and (4) to measured SOX2 and ISL1 expression data, using the same SMAD4 input dynamics described above. Briefly, the values of each parameter must be initialized: we used values found with the previous fit of SOX2 alone, i.e., $\lambda_I = \lambda_S = \lambda$, $\alpha_I = \alpha_S = \alpha$, and $\beta_S = \beta$. We further initialized the inhibition threshold coefficients K_{SI} and K_{IS} at 0.5. We then numerically evaluated the system of ODEs (4) and (5) with those parameters and the SMAD4 inputs described above, and calculated the mean squared error E between the target and calculated expression levels. Then for a set number of iterations, we do the following: perturb the parameters by applying Gaussian noise to each with a variance of 10^{-5} and calculate the new error E_{new} after running the model with the new parameters. If the new error is lower, accept these values as the new parameter values; otherwise, we may still accept the new parameter values with probability $\exp(-\Delta E/k_B T)$, where $\Delta E = E_{\text{new}} - E$, T is the 'effective temperature' for the annealing, and k_B is a tunable constant. The value of T linearly decreases to zero over the course of the iterations so that accepting a set of parameters resulting in a higher cost becomes increasingly unlikely as the algorithm progresses, allowing exploration of the parameter space at early iterations to avoid becoming trapped at a local minimum in the parameter landscape, and settling in to a specific minimum at the end.

The resulting simulated SOX2 dynamics align more closely with the measured dynamics, resolving the discrepancies mentioned above. Furthermore, the relationship between the SMAD4 integral and ISL1 expression produced by the model agrees with the data.

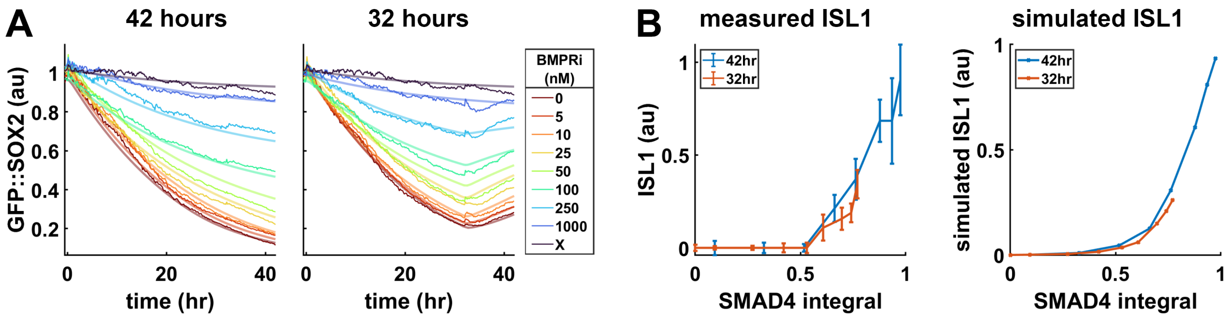

Figure 1: Model simulation with feedback from ISL1. (A) Measured (thick, solid lines) and simulated (thin, semitransparent lines) GFP::SOX2 dynamics over the course of 42 hours differentiation with the indicated treatments for 42 (left) or 32 (right) hours, using the model in (3), (4). (B) Measured (left) and simulated (right) ISL1 level as a function of SMAD4 integral for the same conditions.

The above results were produced with the following parameter values:

Parameter	Value	Meaning	Units
(α_S, α_I)	$(0.0363, 0.09)$	protein dilution + degradation rates	1/hr
β_S	0.0329	constitutive SOX2 production rate	au/hr
(λ_S, λ_I)	$(0.0397, 0.1240)$	coefficients for regulation by SMAD4	1/hr
(n_S, n_I)	$(2, 4)$	Hill function coefficients	N/A
(K_{SI}, K_{IS})	$(0.266, 0.3995)$	inhibition thresholds	au

Inclusion of SOX2 autoregulation

The model in equation (3) predicts a long half-life for SOX2 of around 18h. However, we measured the half-life of SOX2 to be close to 7h (Supplementary Fig. 5I-K), inconsistent with model (3).

Positive autoregulation can slow responses in gene regulatory networks [3]. Given that SOX2 is known to regulate its own production in hPSCs [4], we additionally decided to test whether including positive SOX2 autoregulation would produce the observed longer timescale of differentiation relative to SOX2 half-life. We implemented the positive autoregulation as an additive expression with Hill function reliance on SOX2 of the form

$$\frac{\beta_a[\mathrm{SOX2}]^{n_a}}{K_a^{n_a} + [\mathrm{SOX2}]^{n_a}}$$

so that SOX2 is modeled by

$$\frac{d[\mathrm{SOX2}]}{dt} = \left(\beta - \lambda_S[\mathrm{SMAD4}] + \frac{\beta_a[\mathrm{SOX2}]^{n_a}}{K_a^{n_a} + [\mathrm{SOX2}]^{n_a}}\right) \cdot \frac{K_{SI}^{n_S}}{K_{SI}^{n_S} + [\mathrm{ISL1}]^{n_S}} - \alpha_S[\mathrm{SOX2}]. \qquad (5)$$

We then fit again using simulated annealing, this time fixing α_S to the measured value (Fig.6B), which produced a good fit showing this revised model is consistent with the data.

The parameter values we obtained to generate the results shown in Fig.6BC are:

Parameter	Value	Meaning	Units
(α_S, α_I)	$(0.105, 0.0728)$	protein dilution + degradation rates	1/hr
β	0.0532	constitutive SOX2 production rate	au/hr
β_a	0.0645	SOX2 autoactivation rate	au/hr
(λ_S, λ_I)	$(0.0392, 0.106)$	coefficients for regulation by SMAD4	1/hr
(n_a, n_S, n_I)	$(4, 2, 4)$	Hill function coefficients	N/A
(K_a, K_{SI}, K_{IS})	$(0.765, 0.273, 0.702)$	inhibition thresholds	au

Supplementary Note 2: Algorithm for automated single-cell tracking

Algorithm development

To construct tracks of single cells in time-lapse live-cell microscopy data, we took a "tracking by detection" approach [5], dividing the problem into two steps: (1) segmentation (detection) of all cells in each frame of the time-lapse, and (2) building tracks by linking segmented cells frame-to-frame. A custom single-cell tracking algorithm, based on the approach to particle tracking proposed in [6], and similar to the implementation in the popular Fiji plugin Trackmate [7], was written in MATLAB and integrated into the image processing pipeline.

The approach taken in [6] is to find an approximately optimal solution globally by breaking the tracking problem into two steps. Following this approach, we first link cells one-to-one or one-to-none in consecutive frames, assigning zero or one links from each cell in one frame to cells in the subsequent frame. This is followed by a "gap-closing, merging, splitting" (GMS) step, which addresses common segmentation and linking errors. Gap-closing connects the end of a track in frame t_1 to the beginning of a track in frame $t_2 > t_1 + 1$, and is intended to account for nuclei leaving and re-entering the frame or that fail to be segmented in one or more frames. Merging connects the end of a track to the middle of another track, and accounts for two nuclei in frame t being segmented as a single nucleus in frame $t + 1$. Conversely, splitting connects the beginning of a track in frame t to the middle of a track in a previous frame, and accounts for two nuclei in frame t being segmented as a single nucleus in frame $t-1$ or to a cell dividing in frame $t-1$. These two steps are each cast as a linear assignment problem (LAP), in which a cost is assigned to each possible assignment and the globally optimal solution of the LAP minimizes the sum of possible costs. Fast algorithms have been developed to find the optimal solution for a given cost matrix, so the essential problem is to determine an effective way to assign costs to possible assignments, generally based on the proximity and morphological similarity of nuclei to be linked.

In addition to the general difficulty of robustly tracking through a time-lapse with segmentation errors, an additional challenge is tracking through cell division. To account for this, we modified the approach in Jaqaman to account for both segmentation errors and cell division. To facilitate the identification of dividing cells, we used the object-classification pipeline in Ilastik [8] to label all nuclei as dividing (M-phase, with chromosomes aligned along the metaphase plate immediately prior to cell division) or non-dividing. In the original algorithm, at the frame-frame linking stage each cell in frame t is linked to at most one cell in frame $t + 1$, and splits are only assigned later. We maintain this general framework, looking for only one daughter cell for each cell marked as dividing during frame-frame linking, and aiming to identify the second daughter cell at the merging, splitting, gap closing step. Additionally, the cost function for linking or splitting from

dividing nuclei are modified to facilitate identification of progeny cells, as described below.

In addition to modifying the frame-frame linking and GMS steps to better handle cell divisions, we add a step for merge resolution. This is motivated by the observation that merging events occur purely due to segmentation errors and so our final tracks should not incorporate the merging of two cells into a single object. To resolve merges, we first look for a split from the merged track, indicating that two nuclei moved close together and then apart again, and determine which of the input tracks to the merge more closely matches each of the output tracks from the split. If there is no subsequent split, we assume that either a merge was followed by separation of the two nuclei that failed to be detected as a split, or that the merge was assigned in error. In either case, we determine which input track to the merge more closely matches the track after the merge, and discard the other link.

Frame-frame linking

To link cells in consecutive frames, we define the pairwise linking cost between each cell in frame t and each cell in frame $t+1$, as well as the cost for 'disappearance' of cells from frame t and 'appearance' of cells in frame $t+1$; that is, the cost for a cell in one frame to fail to be linked to any cell in the other. The cost for linking cell i in frame t to cell j in frame $t+1$ is based on the cells' xy positions, as well as the areas and intensities of the nuclei, and whether cell i is marked as dividing. The base cost for linking two cells is the squared euclidean distance between them, given by

$$D_{ij} = (x_i - x_j)^2 + (y_i - y_j)^2$$

To obtain the final cost, we multiply this distance by weights based on the similarity of the two nuclei in area A and intensity I. If we define

$$d_A = \frac{2|A_i - A_j|}{A_i + A_j}, \quad d_I = \frac{2|I_i - I_j|}{I_i + I_j},$$

the the final cost is given by

$$c_{ij} = D_{ij}\,(1 + d_I)\,(1 + d_A)\,.$$

If cell i is labeled as dividing, an additional multiplicative weight is calculated based on the stereotypical rapid movement of daughter nuclei in opposite directions orthogonal to the orientation of the metaphase plate. This weight favors linking to prospective daughter cells found in a direction orthogonal to the metaphase plate. During image processing, the major and minor axes and orientation of an ellipse approximating the nucleus are calculated for each cell, and we use the orientation of cell i's major axis as the orientation of the metaphase plate. We define a normalized vector $\hat{v}$ orthogonal to that orientation. We additionally define the vector pointing from cell i to cell j,

$$\vec{u} = \begin{bmatrix} x_j - x_i \\ y_j - y_i \end{bmatrix},$$

and normalize it to $\hat{u} = \vec{u}/\|\vec{u}\|$. The additional weight for linking cell i to cell j is then

$$w = \frac{3}{2} - |\langle \hat{u}, \hat{v} \rangle|^3,$$

and the resulting overall cost is

$$c_{ij} = w \cdot D_{ij}\,(1 + d_I)\,(1 + d_A)\,.$$

The inner product $\langle \hat{u}, \hat{v} \rangle$ depends on the angle between $\hat{u}$ and $\hat{v}$ and varies from -1 (antiparallel) to 0 (orthogonal) to 1 (parallel). Our weight then varies from 3/2 (orthogonal) to 1/2 (either parallel or antiparallel as daughter cells travel in both directions). Note that the range of values taken by w is unaffected by cubing the inner product, but results in a wider range of angles close to $\pi/2$ producing close to the maximum weight.

We may then construct the cost matrix A with rows corresponding to prospective links from the n_t cells in frame t and columns corresponding to prospective links to the n_{t+1} cells in frame $t+1$, so that $A(i,j) = c_{ij}$, i.e.,

$$A = \begin{bmatrix} c_{11} & \cdots & c_{1n_{t+1}} \\ \vdots & \ddots & \vdots \\ c_{n_t 1} & \cdots & c_{n_t n_{t+1}} \end{bmatrix}.$$

For computational efficiency, we additionally take as an input a maximum linking distance that defines the maximum distance a cell is expected to move between consecutive frames. We treat links between cells at a distance greater than this cutoff as impossible by setting the linking cost to Inf (arbitrarily large). In practice, we used a maximum linking distance of about 15 µm. We additionally define the alternative costs for appearance and disappearance for each cell to be 105% of the maximum finite linking cost. Cost matrices for link rejection are constructed as follows: B_1 is an $n_t \times n_t$ diagonal matrix, with the cost for no link to be made to cell i in frame t at entry $B_1(i,i)$. All off-diagonal entries are set to Inf. Likewise, B_2 is an $n_{t+1} \times n_{t+1}$ diagonal matrix storing the costs to reject links to cells in frame $t+1$ and off-diagonal costs set to Inf. The resulting overall cost matrix is constructed as a block matrix as:

$$C = \begin{bmatrix} A & B_1 \\ B_2 & A^{\mathrm{T}} \end{bmatrix}.$$

Assignments are made by choosing one cost in each row such that no two costs come from the same column and the sum of the costs is minimized. This optimization is performed with the Jonker-Volgenant algorithm for LAPs [9] implemented in MATLAB [10]. Note that the inclusion of the transpose of A in the lower corner ensures that the number of assignments is the same along the rows and columns so that C is a square matrix, and column indices of the assignments in the first n_t rows will match the row indices of the last n_t columns. Likewise, row indices of the assignments to the first n_{t+1} columns will match the column indices of the last n_t rows.

Gap closing, merging, splitting

The GMS step aims to tie up loose ends (and beginnings) from the frame-frame linking step. Track ends are cells without a link to a cell in a subsequent frame and track beginnings are those without a link from a cell in a previous frame (note that these are not mutually exclusive: if a cell has no link in the previous or in the subsequent frame it is both the beginning and end of its own one-cell track). Unlike in frame-frame linking, this step is not local in time and optimizes over possible assignments in the entire time series at once. Each track end is matched to either a track beginning (gap closing), a mid-point of another track (merging), or is given no assignment (track termination). Conversely, each track start is matched to a track end (gap closing), a mid-point of another track (splitting), or is not linked (track initiation). The structure of the cost matrix constructed to handle these possible assignments is more complex, and is constructed as a block

matrix as

$$C = \begin{bmatrix} A_1 & A_2 & A_3 & \text{Inf} \\ B_1 & \text{Inf} & \text{Inf} & B_4 \\ C_1 & \text{Inf} & A_1^{\text{T}} & B_1^{\text{T}} \\ \text{Inf} & D2 & A_2^{\text{T}} & \text{Inf} \end{bmatrix}$$

Here A_1 contains costs for gap closing, A_2 for merging, A_3 for track termination, B_1 for splitting, and C_1 for track initiation. B_4 is a diagonal matrix with costs to reject splits and D_2 likewise has costs to reject merges. As in the frame-frame linking step, the cost matrix C is constructed to be a square matrix with the same possible assignments found along columns as along rows to satisfy the topological structure of the LAP. For instance, it can be seen that the first row of block matrices determines assignments from track ends, as does the third column of block matrices.

In constructing cost matrices, we again impose thresholds for computational efficiency, so links are only considered between cells within a maximum distance δxy_{max} (in practice, about 22.5 um) and a maximum number of time steps apart δt_{max} (in practice, five frames).

The matrix A_1 with costs for gap-closing is similar to the matrix of pairwise linking costs in the frame-frame linking step. Each entry of A_1 stores the cost to link a track end at nucleus i in frame t_1 to a track beginning at nucleus j in frame t_2 with $t_2 > t_1$. If the nuclei are within the threshold distances of one another, $t_2 \leq t_1 + \delta t_{\text{max}}$ and $\|[x_j - x_i, y_j - y_i]^T\| \leq \delta xy_{\text{max}}$, then the cost is given by

$$c_{ij} = \left[(x_i - x_j)^2 + (y_i - y_j)^2 + (t_2 - t_1)^2\right]\left(1 + \frac{|A_i - A_j|}{A_i}\right).$$

As in the frame-frame linking step, if cell i was labeled as dividing, this cost is multiplied by an additional weight w based on the angle between the normal vector to cell i's major axis and the vector between cell i and cell j. Similar to the frame-frame linking step, we build a diagonal cost matrix A_3 to reject links from each track end (cost for track termination) and C_1 to reject links to each track start (cost for track initiation). Like in the frame-frame linking step, these costs are taken to be slightly larger than the maximum finite gap-closing cost.

The matrix A_2 holds costs to merge track ends to midpoints of other tracks, where a track midpoint is any cell that is neither a track end nor a track start, i.e., that has a link both before and after it. Given cell i in frame t_1 that is a track end, we find all track midpoints within the time and distance cutoffs of the track end. For a given midpoint cell j in frame $t_2 \leq t_1 + \delta t_{\text{max}}$, the cost to merge cell i into cell j is given by

$$m_{ij} = \left[(x_i - x_j)^2 + (y_i - y_j)^2\right]\left(1 + \frac{|A_i + A_{j\text{prev}} - A_j|}{A_j}\right),$$

where $A_{j\text{prev}}$ is the area of the nucleus preceding cell j in its track, so that the cost of accepting a merge is lowest when the area of the merged nucleus is the sum of the areas of the two input nuclei. The alternative cost matrix to reject merging holds the cost of rejecting merges for each midpoint for which at least one merge is considered, and is given by

$$b_j = D_{\text{avg}}\left(1 + \frac{|A_{j\text{prev}} - A_j|}{A_j}\right),$$

where D_{avg} is the averaged squared frame-frame displacement for tracks constructed in the frame-frame linking step. Then, the cost for rejecting a merge is lower than the cost of accepting the merge if $|A_{j\text{prev}} - A_j| < |A_{j\text{prev}} + A_i - A_j|$, and if $D_{\text{avg}} < (x_i - x_j)^2 + (y_i - y_j)^2$.

The matrix B_1 holds costs to split track starts from midpoints of other tracks. Similar to the construction of the cost matrix for merging, we take a track start cell i in frame t_1, and find all track midpoints within the time and distance cutoffs. For a given midpoint cell j in frame t_2 with $t_1 > t_2 \geq t_1 - \delta t_{\max}$ that is not marked as dividing, the cost to split cell i from cell j is given by

$$s_{ij} = \left[(x_i - x_j)^2 + (y_i - y_j)^2\right]\left(1 + \frac{|A_i + A_{j\text{next}} - A_j|}{A_j}\right),$$

so the cost is lower if the area before the split is closer to the sum of the areas of the two cells after the split. If cell j is marked as dividing, however, we assume that the first link is to one daughter cell and attempt to find and link to the other daughter cell. The position of the first daughter cell is used to find the expected position of the other, based on the observation that immediately after division, sibling cells move symmetrically away from the location of the parent nucleus prior to division. To find the expected position of the remaining sibling nucleus, the displacement of the first sibling from the parent is found, and the expected position is taken to be at the same displacement but in the opposite direction. The linking cost then uses the distance of each prospective cell from this expected position instead of the distance from the parent cell itself. The resulting linking cost is

$$s_{ij} = \left[(x_i - x_{\text{exp}})^2 + (y_i - y_{\text{exp}})^2\right]\left(1 + \frac{2|A_i - A_{j\text{daughter}}|}{A_i + A_{j\text{daughter}}}\right),$$

Where $A_{j\text{daughter}}$ is the area of the first daughter nucleus in the same frame as the track start. Much like for merging, the alternative cost to reject splits is

$$d_j = D_{\text{avg}}\left(1 + \frac{|A_{j\text{next}} - A_j|}{A_j}\right).$$

After all finite costs have been computed and the aggregate cost matrix is constructed as a block matrix, we numerically optimize to find the best solution to the LAP.

Merge resolution

We resolve merges with the aim of separating the individual tracks that were inputs into the merge at later time points. If a split occurs from the same track soon after a merge (within the maximum time cutoff for gap-closing), and the nucleus from which the split occurred was not labeled as dividing, we assign each input to the merge to one output from the split and discard the links in between; otherwise, one of the links in to the merge is discarded, depending on which input cell bears greater morphological similarity to the cell after the merge. These one or two assignments to resolve each merge are made as a (very small) LAP with costs as in the frame-frame linking step.

Linking live to fixed cells

At the end of live-cell imaging, each sample was fixed and immunofluorescence stained, and we include an additional step to link live cells at the end of the time-series to fixed cells. To ensure a consistent frame of reference, the image of nuclei in the last live frame is aligned to the DAPI stain of the fixed cells with phase correlation-based image registration and the positions of fixed nuclei are adjusted accordingly. Linking of individual cells is done in the same way as the frame-frame linking step during tracking, but with the linking cost based only on distance between nuclei and similarity in area. We do not consider nuclear intensity, which does not necessarily correlate between live data in which nuclei are labeled with fluorescent fusion proteins and fixed data where they are stained with DAPI. Sparse labeling introduces a potential complication to this step: only

10-20% of cells have nuclear markers in the live data, but every cell is stained for DAPI, including those that were not labeled live, so each live nucleus has potentially many more fixed nuclei nearby as candidates to which to link. However, we find that because there is little cell movement in the short time between the end of the live time lapse and the time that cells were fixed, our rigid image registration is robust to only a subset of nuclei being visible in the live data, and the alignment results in corresponding nuclei being very close in the aligned live and fixed data. To prevent erroneous linking to another nearby nucleus in the case that the true fixed nucleus corresponding to a given live cell failed to be properly segmented, we use a reduced maximum linking distance of 10 µm, or about one cell diameter, at this step.

Additional implementation details

The algorithm was implemented in MATLAB, and incorporated into a larger image-processing pipeline.

The the optimal set of assignments for each LAP in the tracking pipeline is computed numerically with a MATLAB implementation [10] of the Jonker-Volgenant algorithm [9].

To account for shifts in the entire field of view between consecutive time points, we implemented a "dejittering" algorithm. We iterated over all frames in the time lapse and at each time loaded a maximal intensity projection of the z stack of images of nuclei at time t_i and t_{i+1} and used phase correlation to determine a global shift between the two images. At each time point, we applied the cumulative shift up to that time to the segmented cell positions in that time, effectively aligning the entire time-lapse to the field of view of the first frame. These updated cell positions are then used in the construction of cost functions for linking during tracking.

Parallelization is used to speed up the algorithm: at the frame-frame linking step, pairs of frames are linked in parallel, and in the gap closing, merging, splitting step, costs are computed in parallel as each block of the cost matrix is constructed.

Supplementary References

1. Levine, M. & Davidson, E. H. Gene regulatory networks for development. en. *Proceedings of the National Academy of Sciences* **102,** 4936–4942. ISSN: 0027-8424, 1091-6490. `https://pnas.org/doi/full/10.1073/pnas.0408031102` (2023) (Apr. 2005).
2. Delás, M. J. & Briscoe, J. en. in *Current Topics in Developmental Biology* 239–266 (Elsevier, 2020). ISBN: 9780128131800. `https://linkinghub.elsevier.com/retrieve/pii/S0070215320300508` (2023).
3. Alon, U. *An introduction to systems biology: design principles of biological circuits* Second edition. en. ISBN: 9781439837177 (CRC Press, Taylor & Francis Group, Boca Raton London New York, 2019).
4. Boyer, L. A. *et al.* Core Transcriptional Regulatory Circuitry in Human Embryonic Stem Cells. en. *Cell* **122,** 947–956. ISSN: 00928674. `https://linkinghub.elsevier.com/retrieve/pii/S0092867405008251` (2023) (Sept. 2005).
5. Magnusson, K. E. G., Jalden, J., Gilbert, P. M. & Blau, H. M. Global Linking of Cell Tracks Using the Viterbi Algorithm. en. *IEEE Transactions on Medical Imaging* **34,** 911–929. ISSN: 0278-0062, 1558-254X. `http://ieeexplore.ieee.org/document/6957576/` (2023) (Apr. 2015).

6. Jaqaman, K. *et al.* Robust single-particle tracking in live-cell time-lapse sequences. en. *Nature Methods* **5,** 695–702. ISSN: 1548-7091, 1548-7105. `http://www.nature.com/articles/nmeth.1237` (2022) (Aug. 2008).
7. Tinevez, J.-Y. *et al.* TrackMate: An open and extensible platform for single-particle tracking. en. *Methods* **115,** 80–90. ISSN: 10462023. `https://linkinghub.elsevier.com/retrieve/pii/S1046202316303346` (2023) (Feb. 2017).
8. Berg, S. *et al.* ilastik: interactive machine learning for (bio)image analysis. en. *Nature Methods* **16,** 1226–1232. ISSN: 1548-7091, 1548-7105. `http://www.nature.com/articles/s41592-019-0582-9` (2022) (Dec. 2019).
9. Jonker, R. & Volgenant, A. A shortest augmenting path algorithm for dense and sparse linear assignment problems. en. *Computing* **38,** 325–340 (1987).
10. Cao, L. *LAPJV - Jonker-Volgenant Algorithm for Linear Assignment Problem V3.0* `https://www.mathworks.com/matlabcentral/fileexchange/26836-lapjv-jonker-volgenant-algorithm-for-linear-assignment-problem-v3-0` (2023).
