# Supplementary material for: Time-integrated BMP signaling determines fate in a stem cell model for early human development
Source: Nat Commun. 2024 Feb 17;15:1471. doi: 10.1038/s41467-024-45719-9 (PMC10874454; doi:10.1038/s41467-024-45719-9)
Supplement: Supplementary file 3 — Description of Additional Supplementary Files [file 41467_2024_45719_MOESM3_ESM.pdf]

Description of Additional Supplementary Files

File Name: Supplementary Data 1
Description: List of clustered transcripts from our bulk RNA sequencing analysis, labeled with cluster id, correlation with SMAD4, slope of a linear fit with SMAD4, and whether each gene is marked as a candidate integrator.
