# Supplementary material for: Time-integrated BMP signaling determines fate in a stem cell model for early human development
Source: Nat Commun. 2024 Feb 17;15:1471. doi: 10.1038/s41467-024-45719-9 (PMC10874454; doi:10.1038/s41467-024-45719-9)
Supplement: Supplementary file 5 — Reporting Summary [file 41467_2024_45719_MOESM5_ESM.pdf]

nature portfolio

Corresponding author(s): Idse Heemskerk
iheemske@umich.edu

Last updated by author(s): Jan 23, 2024

Reporting Summary

Nature Portfolio wishes to improve the reproducibility of the work that we publish. This form provides structure for consistency and transparency in reporting. For further information on Nature Portfolio policies, see our Editorial Policies and the Editorial Policy Checklist.

Statistics

For all statistical analyses, confirm that the following items are present in the figure legend, table legend, main text, or Methods section.

n/a	Confirmed	
☐	☒	The exact sample size (*n*) for each experimental group/condition, given as a discrete number and unit of measurement
☐	☒	A statement on whether measurements were taken from distinct samples or whether the same sample was measured repeatedly
☐	☒	The statistical test(s) used AND whether they are one- or two-sided<br>*Only common tests should be described solely by name; describe more complex techniques in the Methods section.*
☒	☐	A description of all covariates tested
☒	☐	A description of any assumptions or corrections, such as tests of normality and adjustment for multiple comparisons
☐	☒	A full description of the statistical parameters including central tendency (e.g. means) or other basic estimates (e.g. regression coefficient) AND variation (e.g. standard deviation) or associated estimates of uncertainty (e.g. confidence intervals)
☒	☐	For null hypothesis testing, the test statistic (e.g. *F*, *t*, *r*) with confidence intervals, effect sizes, degrees of freedom and *P* value noted<br>*Give P values as exact values whenever suitable.*
☒	☐	For Bayesian analysis, information on the choice of priors and Markov chain Monte Carlo settings
☒	☐	For hierarchical and complex designs, identification of the appropriate level for tests and full reporting of outcomes
☐	☒	Estimates of effect sizes (e.g. Cohen's *d*, Pearson's *r*), indicating how they were calculated

Our web collection on statistics for biologists contains articles on many of the points above.

Software and code

Policy information about availability of computer code

Data collection	Microscopy images were collected with an Andor Dragonfly spinning disk confocal microscope using Andor Fusion software version 2.3.0.31, or with a Nikon/Yokogawa spinning disk confocal microscope using NIS Elements AR software version 5.41.02. Simulated expression data with our mathematical model was generated with custom code written in MATLAB (versions 2019b to 2023a), available in the public repository at https://github.com/idse/BMPintegral or on Zenodo at https://doi.org/10.5281/zenodo.10076773.
Data analysis	Microscopy data was processed and quantified with a custom image-processing pipeline written in MATLAB (2019b - 2023a), with code publicly available at https://github.com/idse/BMPintegral or https://doi.org/10.5281/zenodo.10076773. Segmentation of nuclei was done with Ilastik (v1.3.3post2) and Cellpose (v1) and segmentation of cell bodies was done using Ilastik. Code written in MATLAB and available in the above repository was used for downstream analysis of quantified microscopy data, single-cell tracking, simulation of the mathematical model, and generation of figures. Denoising of single-cell signaling histories was done in Python (v3.8) with MAGIC (Markov Affinity-based Graph Imputation of Cells)

For manuscripts utilizing custom algorithms or software that are central to the research but not yet described in published literature, software must be made available to editors and reviewers. We strongly encourage code deposition in a community repository (e.g. GitHub). See the Nature Portfolio guidelines for submitting code & software for further information.

Data

Policy information about availability of data

All manuscripts must include a data availability statement. This statement should provide the following information, where applicable:

- Accession codes, unique identifiers, or web links for publicly available datasets
- A description of any restrictions on data availability
- For clinical datasets or third party data, please ensure that the statement adheres to our policy

Processed data and corresponding code used to make figures in the manuscript are provided at https://github.com/idse/BMPintegral. RNA sequencing data has been deposited in GEO under accession number GSE229675 [https://www.ncbi.nlm.nih.gov/geo/query/acc.cgi?acc=GSE229675]. Source data are provided with this paper. Raw image data will be made available upon reasonable request but are too large to practically host in a public data repository.

Research involving human participants, their data, or biological material

Policy information about studies with human participants or human data. See also policy information about sex, gender (identity/presentation), and sexual orientation and race, ethnicity and racism.

Reporting on sex and gender	*Use the terms sex (biological attribute) and gender (shaped by social and cultural circumstances) carefully in order to avoid confusing both terms. Indicate if findings apply to only one sex or gender; describe whether sex and gender were considered in study design; whether sex and/or gender was determined based on self-reporting or assigned and methods used. Provide in the source data disaggregated sex and gender data, where this information has been collected, and if consent has been obtained for sharing of individual-level data; provide overall numbers in this Reporting Summary. Please state if this information has not been collected. Report sex- and gender-based analyses where performed, justify reasons for lack of sex- and gender-based analysis.*
Reporting on race, ethnicity, or other socially relevant groupings	*Please specify the socially constructed or socially relevant categorization variable(s) used in your manuscript and explain why they were used. Please note that such variables should not be used as proxies for other socially constructed/relevant variables (for example, race/ethnicity should not be used as a proxy for socioeconomic status). Provide clear definitions of the relevant terms used, how they were provided (by the participants/respondents, the researchers, or third parties), and the method(s) used to classify people into the different categories (e.g. self-report, census or administrative data, social media data, etc.) Please provide details about how you controlled for confounding variables in your analyses.*
Population characteristics	*Describe the covariate-relevant population characteristics of the human research participants (e.g. age, genotypic information, past and current diagnosis and treatment categories). If you filled out the behavioural & social sciences study design questions and have nothing to add here, write "See above."*
Recruitment	*Describe how participants were recruited. Outline any potential self-selection bias or other biases that may be present and how these are likely to impact results.*
Ethics oversight	*Identify the organization(s) that approved the study protocol.*

Note that full information on the approval of the study protocol must also be provided in the manuscript.

Field-specific reporting

Please select the one below that is the best fit for your research. If you are not sure, read the appropriate sections before making your selection.

[x] Life sciences [ ] Behavioural & social sciences [ ] Ecological, evolutionary & environmental sciences

For a reference copy of the document with all sections, see nature.com/documents/nr-reporting-summary-flat.pdf

Life sciences study design

All studies must disclose on these points even when the disclosure is negative.

Sample size	Sample sizes were not predetermined. For experiments with micropatterned colonies, we analyzed n = 3 to 5 colonies per condition based on previous experience and published data confirming the colony-to-colony reproducibility of results in this system. For microscopy in disordered culture based on population averages, we took at least four replicate images per condition, generally containing several hundred cells per image, again based on previous experience. Note that an image is not an intrinsic unit of data, and the amount of data captured by a single field of view depends on, among other things, the objective magnification and size of the camera sensor, so that a single replicate could effectively be split into multiple non-overlapping fields of view, each of which could just as well be considered a separate sample. For single-cell tracking experiments, several hundred to nearly one thousand cells were processed each as an individual sample, depending on the number that could be successfully tracked.
Data exclusions	In figure 4, micropatterned colonies with unusually low seeding density which collapsed or failed to form a normal circular morphology were excluded from analysis which used averages based on distance of cells from the colony edge. In image analysis, quantification of signaling and gene expression was done only for cells in interphase, as the morphology of dividing nuclei and their boundaries with the cytosol could not be accurately measured based on images of fluorescently tagged histones or DAPI staining, and not all transcription factors remain bound to

	condensed chromatin.
Replication	Most experiments were performed at least twice and all attempts at replication were successful.
Randomization	In differentiation experiments, genetically identical cells were randomly allocated between conditions upon seeding. Imaging positions were randomly chosen when collecting microscopy data.
Blinding	Blinding with respect to cell or genotype identity was not relevant to this study, as each condition contained genetically identical cells in all experiments. The investigators could not be blinded to culture or treatment conditions during experiments.

Reporting for specific materials, systems and methods

We require information from authors about some types of materials, experimental systems and methods used in many studies. Here, indicate whether each material, system or method listed is relevant to your study. If you are not sure if a list item applies to your research, read the appropriate section before selecting a response.

Materials & experimental systems

n/a	Involved in the study	
☐	☒	Antibodies
☐	☒	Eukaryotic cell lines
☒	☐	Palaeontology and archaeology
☒	☐	Animals and other organisms
☒	☐	Clinical data
☒	☐	Dual use research of concern
☒	☐	Plants

Methods

n/a	Involved in the study	
☒	☐	ChIP-seq
☒	☐	Flow cytometry
☒	☐	MRI-based neuroimaging

Antibodies

Antibodies used	mouse anti-ISL1 (DSHB, Cat# 39.4D5, 1:200), rabbit anti-SOX2 (Cell Signaling Technology, cat# 3579S, 1:200), goat anti-NANOG (R&D Systems, cat# AF1997, 1:100), goat anti-HAND1 (R&D Systems, cat# AF3168, 1:200), rabbit anti-GATA3 (Cell Signaling Technology, cat# 5852S, 1:800), mouse anti-TFAP2C (Santa Cruz Biotechnology, cat# SC-12762, 1:150), mouse anti-OCT3/4 (BD Biosciences, cat# 611202, 1:400), rabbit anti-pSmad1/5/9 (Cell Signaling Technology, cat# 13820S, 1:100), mouse anti-Smad2/3 (BD Biosciences, cat# 610843, 1:100), goat anti-Brachyury (R&D Systems, cat# AF2085, 1:300)
Validation	mouse anti-ISL1 (https://dshb.biology.uiowa.edu/39-4D5)<br>rabbit anti-SOX2 (https://www.cellsignal.com/products/primary-antibodies/sox2-d6d9-xp-rabbit-mab/3579)<br>goat anti-NANOG (https://www.rndsystems.com/products/human-nanog-antibody_af1997)<br>goat anti-HAND1 (https://www.rndsystems.com/products/human-hand1-antibody_af3168)<br>rabbit anti-GATA3 (https://www.cellsignal.com/products/primary-antibodies/gata-3-d13c9-xp-rabbit-mab/5852)<br>mouse anti-TFAP2C (https://www.scbt.com/p/ap-2gamma-antibody-6e4-4)<br>mouse anti-OCT3/4 (https://www.bdbiosciences.com/en-us/products/reagents/microscopy-imaging-reagents/immunofluorescence-reagents/purified-mouse-anti-oct3-4.611202)<br>rabbit anti-pSmad1/5/9 (https://www.cellsignal.com/products/primary-antibodies/phospho-smad1-ser463-465-smad5-ser463-465-smad9-ser465-467-d5b10-rabbit-mab/13820)<br>mouse anti-Smad2/3 (https://www.bdbiosciences.com/en-us/products/reagents/microscopy-imaging-reagents/immunofluorescence-reagents/purified-mouse-anti-smad2-3.610843)<br>goat anti-Brachyury (https://www.rndsystems.com/products/human-mouse-brachyury-antibody_af2085)

Eukaryotic cell lines

Policy information about cell lines and Sex and Gender in Research

Cell line source(s)	ESI017 (XX) embryonic stem cells were obtained from ESI BIO. RUES2 (XX), RUES2 GFP::SMAD4, and RUES2 RFP::SMAD1 embryonic cells were a gift of Ali Brivanlou. WTC11 GFP::SOX2 induced pluripotent cells (XY) were produced by the Allen Institute and obtained from the Coriell Institute. ESI017 tetO-SOX2 were produced from parent ESI017 (XX) cells in this work.
Authentication	Cells were grown in mTeSR1 to maintain pluripotency, and regularly checked for pluripotent morphology. Cells were additionally immunostained for pluripotency markers (OCT4, SOX2, NANOG) to confirm pluripotent identity, and a stain for SOX2 and NANOG confirmed pluripotency in a subset of cells in nearly every experiment.
Mycoplasma contamination	Cells were regularly tested and found negative for mycoplasma contamination.
Commonly misidentified lines<br>(See ICLAC register)	None
